# Exosome-like nanovesicles derived from kale juice enhance collagen production by downregulating Smad7 in human skin fibroblasts

**DOI:** 10.3389/fnut.2025.1486572

**Published:** 2025-02-10

**Authors:** Peihan Hsu, Yuriko Kamijyo, Emiri Koike, Saki Ichikawa, Yifeng Zheng, Tomohiro Ohno, Shigeru Katayama

**Affiliations:** ^1^Department of Agriculture, Graduate School of Science and Technology, Shinshu University, Nagano, Japan; ^2^Institute for Biomedical Sciences, Interdisciplinary Cluster for Cutting Edge Research, Shinshu University, Nagano, Japan; ^3^Yakult Health Foods Co., Ltd., Oita, Japan

**Keywords:** collagen, exosomes, exosome-like nanovesicles, fibroblasts, kale, miRNA

## Abstract

Plant-derived exosome-like nanovesicles (ELNs) are critical mediators of cross-kingdom communication, modulating gene expression in animal cells despite their plant origin. In this study, we investigated the effects of glucoraphanin-enriched kale (GEK)-derived ELNs (GELNs) on collagen production in normal human dermal fibroblasts NB1RGB. The ELNs isolated from GEK juice powder had particle sizes similar to those of typical exosomes. GELNs increased type I collagen expression in NB1RGB cells significantly. Microarray analysis demonstrated that GELN-derived total RNA upregulated the expression of genes related to extracellular matrix formation, including those involved in collagen synthesis. Further investigation revealed that microRNA-enriched fraction of GELNs promoted collagen production by inhibiting the expression of Smad7. These findings suggest that GELNs and their microRNA content enhance collagen production through the downregulation of Smad7.

## 1 Introduction

Exosomes are endosome-derived extracellular vesicles, approximately 30–150 nm in diameter (1, 2). They are released by cells and carry various biomolecules, including proteins, lipids, mRNA, and non-coding RNA, enclosed within a lipid bilayer membrane that provides protection and enables their transport. Extensive research has shown that exosomes play an important role in cellular communication both locally and over long distances (3). Plants secrete exosome-like nanovesicles (ELNs) similar to mammalian exosomes, which play crucial roles in the plant immune system and defense responses (4). ELNs can transport various bioactive substances, including nucleic acids, proteins, vitamins, antioxidant molecules, and metabolites. Plant-derived ELNs play vital roles in cross-kingdom communication, impacting gene expression in animal cells (5–8). MicroRNAs (miRNAs) are functional non-coding RNA that are not translated into proteins. miRNAs bind to mRNA within cells in a base-complementary (or partially complementary) manner and regulate gene expression. Xiao et al. identified 418 miRNAs, primarily 20–22 nt in size, from ELNs isolated from 11 edible fruits and vegetables (8). In addition, certain miRNAs have been predicted to target inflammatory genes: miR-5781 in soybean-derived ELNs specifically targeted interleukin-17A, which is involved in inflammatory responses.

Kale (*Brassica oleracea* var. *acephala*) is a green leafy vegetable rich in bioactive compounds, which provide health benefits, including carotenoids, flavonoids, and glucosinolates (9–11). Glucoraphanin, the precursor of sulforaphane, is an important functional glucosinolate widely found in *Brassica* vegetables (12). Sulforaphane is a potent activator of nuclear factor erythroid 2-related factor 2 (Nrf2), which induces phase II detoxifying and antioxidant enzymes (13). However, sulforaphane is highly unstable when exposed to heat, light, or oxidants (14). In contrast, glucoraphanin is stable and can be converted into sulforaphane by myrosinase or intestinal bacteria assimilation (15). Recently, glucoraphanin-enriched kale (GEK) has been developed as a new cultivar, allowing stable *in vivo* utilization of the potent activity of sulforaphane. A randomized, double-blind, placebo-controlled clinical trial demonstrated that GEK intake increased skin moisture and improved skin elasticity, particularly in participants aged 40 years and older (16). We previously demonstrated that long-term GEK intake suppresses skin aging in senescence-accelerated mouse prone 1 (SAMP1) (17). In addition, the GEK-treated mouse group showed upregulation of antioxidant enzymes and promotion of collagen production induced by Nrf2 activation and the transforming growth factor (TGF)-*β*/Smad signaling pathway, respectively. Nrf2 activation, which leads to the upregulation of antioxidant enzymes, is attributed to the conversion of glucoraphanin to sulforaphane following ingestion. However, the specific components of GEK responsible for promoting collagen production remain unknown.

Plant-derived ELNs containing miRNAs have the potential to exhibit cross-kingdom regulation. Based on this finding, we hypothesized that GEK-derived ELNs (GELNs) enhance collagen production, with the miRNAs within these ELNs being active components. We isolated ELNs from GEK to test this hypothesis and investigated whether they could enhance collagen production in human dermal fibroblasts. Additionally, we extracted total RNA, including miRNAs, to analyze the expression of collagen and extracellular matrix components. We also examined the specific effects of the miRNA-enriched fraction isolated from GELNs.

## 2 Methods

### 2.1 Isolation and characterization of GELNs

Spray-dried GEK juice powder, consisting of kale juice and dextrin in a 1:1.5 ratio, was obtained from Yakult Health Food Company (Oita, Japan). GELNs were isolated using differential centrifugation and ultracentrifugation. Briefly, GEK juice powder (1.5 g) was added to 15 mL phosphate-buffered saline (PBS). The suspension was centrifugated at 4°C: 1,200 × g for 20 min, 3,000 × g for 20 min, and 10,000 × g for 60 min to remove the coarse particles. The supernatant was filtered through a 0.45 μm filter and centrifuged at 90,000 × g for 120 min. The resultant pellet was resuspended in PBS and stored at −80°C until use. The size and concentration of the GELNs were analyzed using a NanoSight LM10 (Malvern Panalytical, UK) in a laboratory at FUJIFILM Wako Pure Chemical Corporation (Osaka, Japan). GELNs were identified using transmission electron microscopy (TEM) analysis. Briefly, the GELNs were fixed with glutaraldehyde and stained with phosphotungstic acid using a carbon/Formvar film-coated TEM grid (Alliance Biosystems, Osaka, Japan). The samples were then imaged using a TEM (JEM-1400, JEOL, Tokyo, Japan).

### 2.2 Extraction of total RNA and miRNA-enriched fraction

Total RNA, including miRNA, and the miRNA-enriched fraction were extracted from GELNs using the miRNeasy Mini kit (Qiagen, Germantown, MD, USA) and the Nucleospin miRNA kit (Macherey-Nagel, Germany), referred to as GELN-RNA and GELN-miRNA, respectively, according to the manufacturer’s instructions. RNA purity and concentration were assessed using a NanoDrop Lite spectrophotometer (Thermo Scientific, Waltham, MA, USA).

### 2.3 Cell culture

NB1RGB cells were cultured in minimum essential medium *α* (MEM-α) containing 10% fetal bovine serum, 100 U/mL penicillin, and 100 μg/mL streptomycin at 37°C in a humidified incubator with 5% CO_2_. The cells were seeded into 12-well plates at a density of 2.5 × 10^5^ cells/well and cultured for 24 h. Subsequently, the cells were incubated with GELNs. At 24 h post-treatment, the cells were collected for western blotting. At 48 h post-treatment, the culture supernatants were collected to determine type I collagen and hyaluronic acid levels.

### 2.4 Western blot analysis

We extracted total protein from the cells using a radioimmunoprecipitation assay buffer (Santa Cruz Biotechnology, CA, USA). Protein concentrations were determined using a Protein Assay BCA kit (FUJIFILM Wako Pure Chemical). Equal amounts of protein (7 μg) were separated by sodium dodecyl sulfate-polyacrylamide gel electrophoresis and transferred onto polyvinylidene fluoride (PVDF) membranes. The PVDF membranes were blocked with 5% skim milk for 1 h (collagen I and *β*-actin) or with Blocking One (Nacalai Tesque, Kyoto, Japan) for 45 min (Smad7 and β-actin). The membranes were incubated with the respective primary antibodies overnight at 4°C, followed by incubation with horseradish peroxidase (HRP)-conjugated secondary antibodies for 1 h. The antibodies used were collagen I (1:5000; Abcam, Cambridge, UK), Smad7 (1:1000; Santa Cruz Biotechnology), and HRP-conjugated *β*-actin (1:5000, Cell Signaling, Danvers, MA, USA). The proteins were visualized using EzWestLumi Plus reagent (ATTO, Tokyo, Japan), and chemiluminescent signals were detected using Amersham ImageQuant 800 (Cytiva, Tokyo, Japan). The relative abundances of the proteins were determined using ImageJ software (National Institutes of Health, Bethesda, MD, USA) with normalization relative to *β*-actin.

### 2.5 RNA transfection

GELN-RNA, GELN-miRNA, synthetic mimic miRNA (Horizon Discovery, United Kingdom), or negative control miRNA (Bioneer, Korea) were transfected into NB1RGB cells using Xfect RNA Transfection Reagent (Takara Bio) according to the manufacturer’s protocol. Briefly, NB1RGB cells were plated in 12-well microplates at a density of 2.5 × 10^5^ cells/well and pre-incubated for 24 h. Transfection was performed using 1.0 μg/mL of GELN-RNA, 1.0 μg/mL of GELN-miRNA, 5 pmol/mL of synthetic mimic miRNA, or 5 pmol/mL of control miRNA, along with 5 μL/mL of Xfect RNA Transfection Reagent. Cells were incubated for 4 h following transfection. Then, the medium was replaced with serum-free media. Following transfection, the cells were harvested at 12 h for quantitative polymerase chain reaction (qPCR), at 24 h for western blotting, and at 48 h for the measurement of type I collagen and hyaluronic acid levels.

### 2.6 qPCR

Total RNA was isolated from cells using a miRNeasy Mini kit (Qiagen), and cDNA synthesis was conducted using a ReverTra Ace qPCR RT Master Mix with gDNA Remover (TOYOBO) according to the manufacturer’s instructions. Next, qPCR was performed using the THUNDERBIRD SYBR qPCR Mix (TOYOBO) on a TP850 Thermal Cycler Dice Real Time System (Takara Bio). The primer sequences are summarized in Supplementary Table 1. The primers were purchased from Eurofins Genomics (Tokyo, Japan). Data were analyzed using the 2^−ΔΔCt^ method and presented as the fold change in gene expression normalized to 18S ribosomal mRNA levels relative to the controls.

### 2.7 DNA microarray analysis

Gene expression profiles were examined using DNA microarray analysis. Total RNA was isolated from the cells treated with GELN-RNA for 12 h using a miRNeasy Mini kit (Qiagen) according to the manufacturer’s instructions. RNA samples were prepared from three independent biological replicates (*n* = 3). DNA microarray analysis was performed using the human Clariom D array (Thermo Fisher Scientific) by Filgen. RNA quality and quantity were assessed using an Agilent 2,100 Bioanalyzer (Agilent Technologies). Gene Ontology (GO) and pathway analyses were performed using a Filgen Microarray Data Analysis Tool (version 3.2). GO analysis primarily describes three terms: “biological process,” “molecular function,” and “cellular component.” The *p*-value for each GO term and pathway was calculated using the two-tailed Fisher’s exact test. Differentially expressed genes were identified with *p*-value < 0.05 and a fold change of >1.8 or < 0.556. GO terms or pathways with *p*-value < 0.05 were considered statistically significant.

### 2.8 Measurement of collagen and hyaluronic acid production

The levels of collagen and hyaluronic acid in the culture media were measured using a Human Collagen Type I enzyme-linked immunosorbent assay (ELISA) Kit (ACEL, Kanagawa, Japan) and a Hyaluronan Quantification Kit (Cosmo Bio, Tokyo, Japan), respectively.

### 2.9 Statistical analysis

The results are expressed as the mean ± standard deviation. We evaluated the statistical significance of the difference between the experimental and corresponding control groups using a one-way analysis of variance, followed by Dunnett’s test versus the control group for comparisons among three or more groups, Tukey’s multiple comparison test for comparisons among all groups, or a Student’s t-test for comparisons between two groups performed using GraphPad Prism 10. All comparisons were made as specified, and differences were considered statistically significant at *p* < 0.05.

## 3 Results

### 3.1 Characterization of GELNs

**Figure 1:**
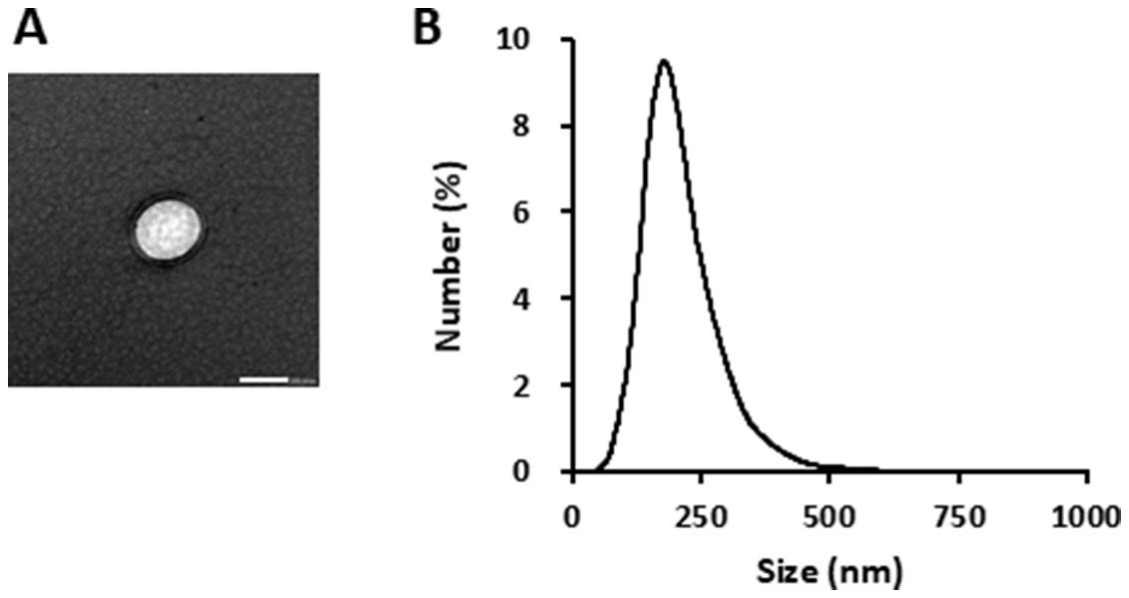
Characterization of glucoraphanin-enriched kale-derived exosome-like nanovesicles (GELNs). **(A)** Transmission electron microscopy image of GELNs. Scale bar = 200 nm. **(B)** Nanoparticle size analyzer measurement of the particle size of GELNs.

TEM observations indicated that the GELNs were negatively stained and spherical particles (Figure 1A). The average particle size was 212.0 ± 4.9 nm, and the main particle size peak was 181.6 nm, indicating that GEK juice contains ELNs (Figure 1B).

### 3.2 Effect of GELNs on collagen expression in NB1RGB cells

**Figure 2:**
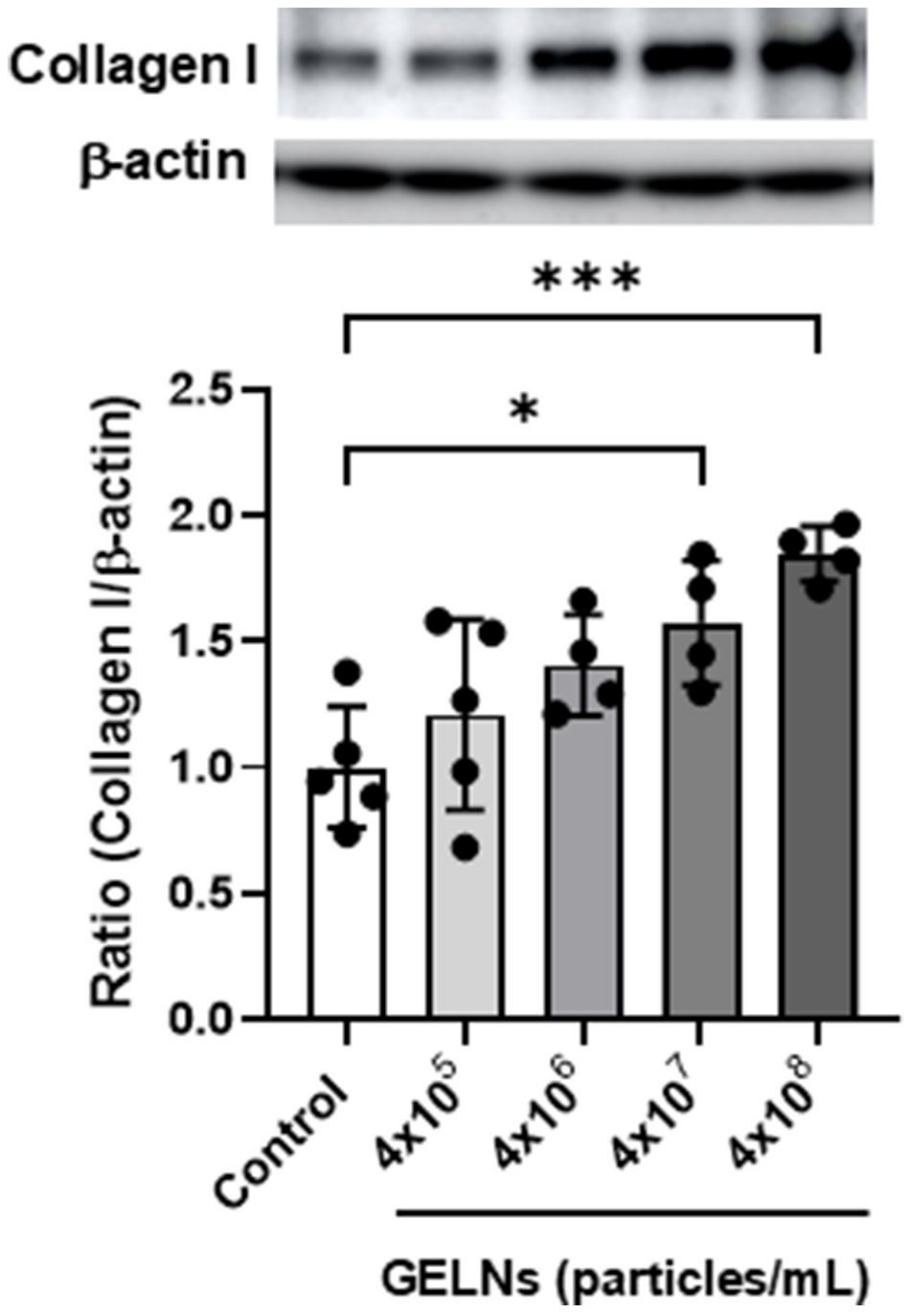
Effects of glucoraphanin-enriched kale-derived exosome-like nanovesicles (GELNs) on collagen I expression in NB1RGB cells. Values are presented as mean ± standard deviation (*n* = 4–5). Significant differences between groups were analyzed using one-way analysis of variance, followed by Dunnett’s test. *p < 0.05, ***p < 0.001 compared to the control.

**Figure 3:**
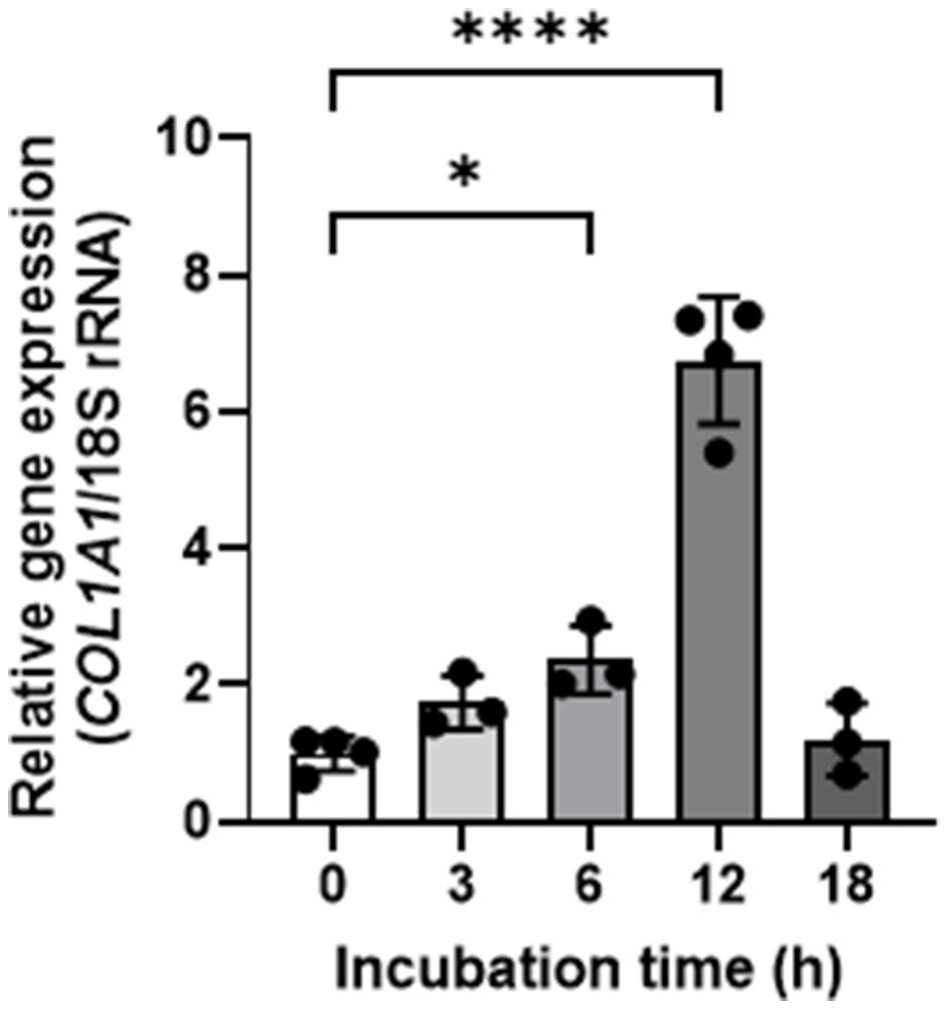
Effects of glucoraphanin-enriched kale-derived exosome-like nanovesicles on *COL1A1* gene expression in NB1RGB cells. Values are presented as mean ± standard deviation (*n* = 3–4). Significant differences between groups were analyzed using one-way analysis of variance, followed by Dunnett’s test. *p < 0.05, ****p < 0.0001 compared to 0 h.

We evaluated the effect of GELNs on type I collagen expression in normal skin fibroblasts NB1RGB. GELN treatment resulted in a dose-dependent increase in type I collagen expression compared with the control group (Figure 2). To explore the underlying mechanism, we hypothesized that RNA encapsulated within GELNs induces type I collagen expression and investigated the effects of transfection with GELN-RNA. The gene expression level of *COL1A1* (encoding type I collagen α1 chain) increased over time with transfection with GELN-RNA, reaching its maximum value at 12 h (Figure 3).

### 3.3 Effect of GELN-RNA on gene expression in NB1RGB cells

**Figure 4:**
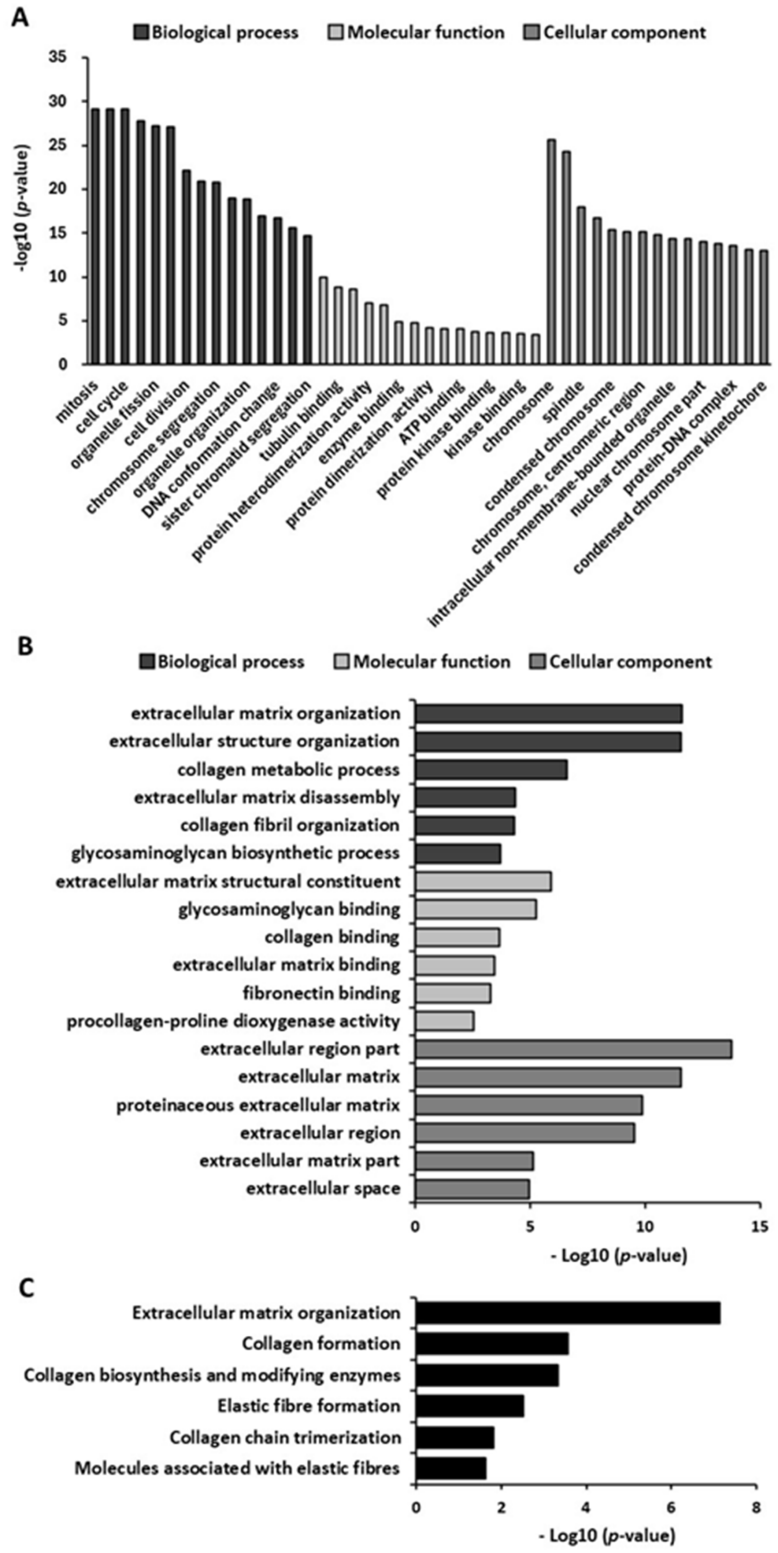
Gene Ontology (GO) and pathway enrichment analysis of upregulated genes (*n* = 3). **(A)** Bar chart of top 15 GO enriched terms in biological processes, molecular function, and cellular component. **(B)** Bar chart of GO terms related to extracellular matrix formation-related GO terms. **(C)** Bar chart of pathways related to extracellular matrix formation.

We evaluated the potential of GELN-RNA to induce the expression of genes involved in extracellular matrix formation using microarray analysis. GELN-RNA treatment resulted in the upregulation and downregulation of 957 and 1,003 genes, respectively. GO enrichment analysis showed that the top 15 biological processes significantly upregulated in GELN-RNA-treated cells were primarily related to cell cycle processes, including mitosis, nuclear division, and chromosome segregation (Figure 4A). The top 15 molecular functions included activities related to microtubule binding, protein binding, and extracellular matrix structural constituents. The top 15 cellular components were largely associated with chromosomal and spindle structures and components of the microtubule cytoskeleton. These findings suggest that GELN-RNA influences cellular processes involved in cell cycle regulation and the structural organization of the extracellular matrix.

Figure 4B shows the significantly upregulated biological processes related to extracellular matrix formation in GELN-RNA-treated cells. These include processes related to extracellular matrix organization and remodeling, collagen metabolism, and glycosaminoglycan biosynthesis. These findings suggest that GELN-RNA influences the cellular processes involved in the structural and functional organization of the extracellular matrix, which may affect tissue remodeling and repair. The enriched molecular functions in GELN-RNA-treated cells included extracellular matrix interactions, particularly in structural constituents, and binding activities related to glycosaminoglycan, collagen, fibronectin, and collagen modification enzymes. Furthermore, the enriched cellular components are involved in the extracellular environment, encompassing various regions of the extracellular matrix. Pathway analysis revealed significant alterations in extracellular matrix organization and formation pathways, such as collagen biosynthesis and modification, elastic fiber formation, collagen trimerization, and the involvement of molecules associated with elastic fibers (Figure 4C).

**Figure 5:**
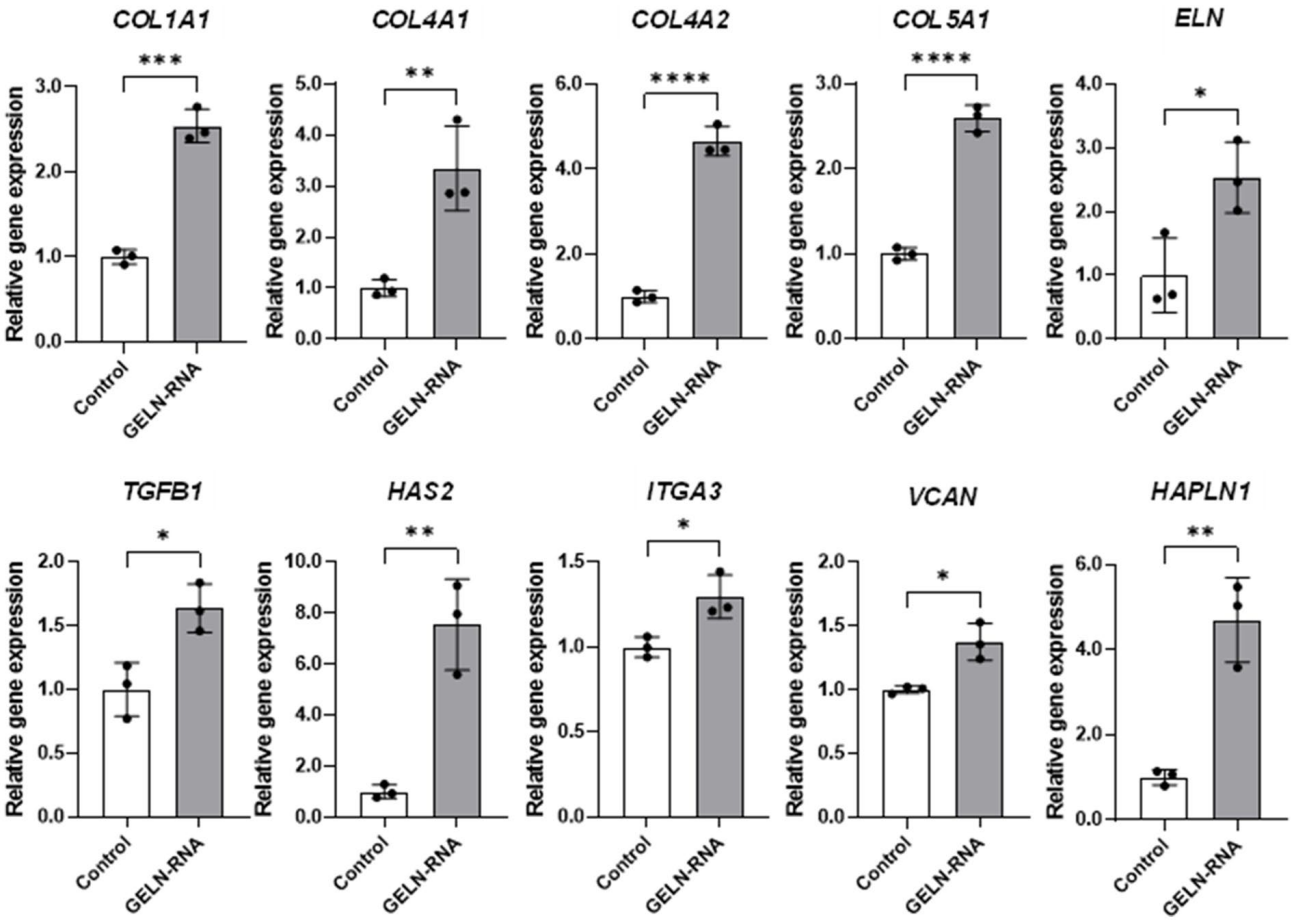
Quantitative PCR validation of differentially expressed genes involved in extracellular matrix formation. Values are presented as mean ± standard deviation (*n* = 3). Significant differences between groups were analyzed using Student’s *t*-test. *p < 0.05, **p < 0.01, ***p < 0.001, ****p < 0.0001 compared to the control.

We validated the transcriptional changes in some of these genes using qPCR to confirm the effect of GELN-RNA treatment on the observed gene expression levels. Significant increases in the mRNA expression of these genes were observed in the cells transfected with GELN-RNA (Figure 5).

### 3.4 Effects of GELN-RNA on the production of collagen and hyaluronic acid in NB1RGB cells

**Figure 6:**
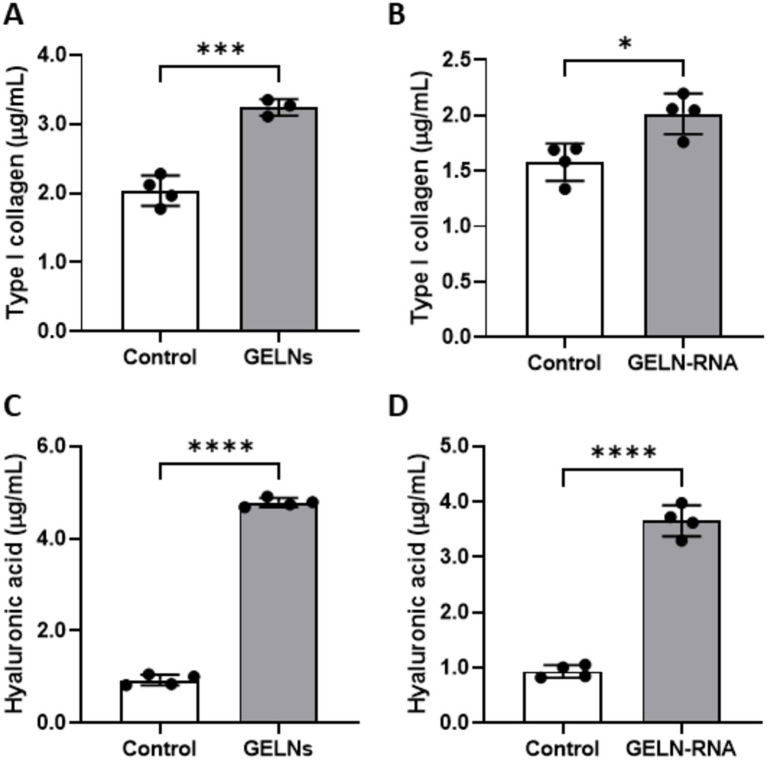
Effects of glucoraphanin-enriched kale-derived exosome-like nanovesicles (GELNs) **(A,C)** and GELN-RNA **(B,D)** on collagen **(A,B)** and hyaluronic acid **(C,D)** production in NB1RGB cells. Values are presented as mean ± standard deviation (*n* = 3–4). Significant differences between groups were analyzed using Student’s *t*-test. *p < 0.05, ***p < 0.001, ****p < 0.0001 compared to the control.

The levels of type I collagen in the NB1RGB culture medium increased following both GELNs and GELN-RNA treatments (Figures 6A,B). A similar increase was observed in the production of hyaluronic acid in NB1RGB cells treated with GELNs or GELN-RNA (Figures 6C,D).

### 3.5 Effects of miRNA encapsulated in GELNs on type I collagen and Smad7 expression in NB1RGB cells

**Figure 7:**
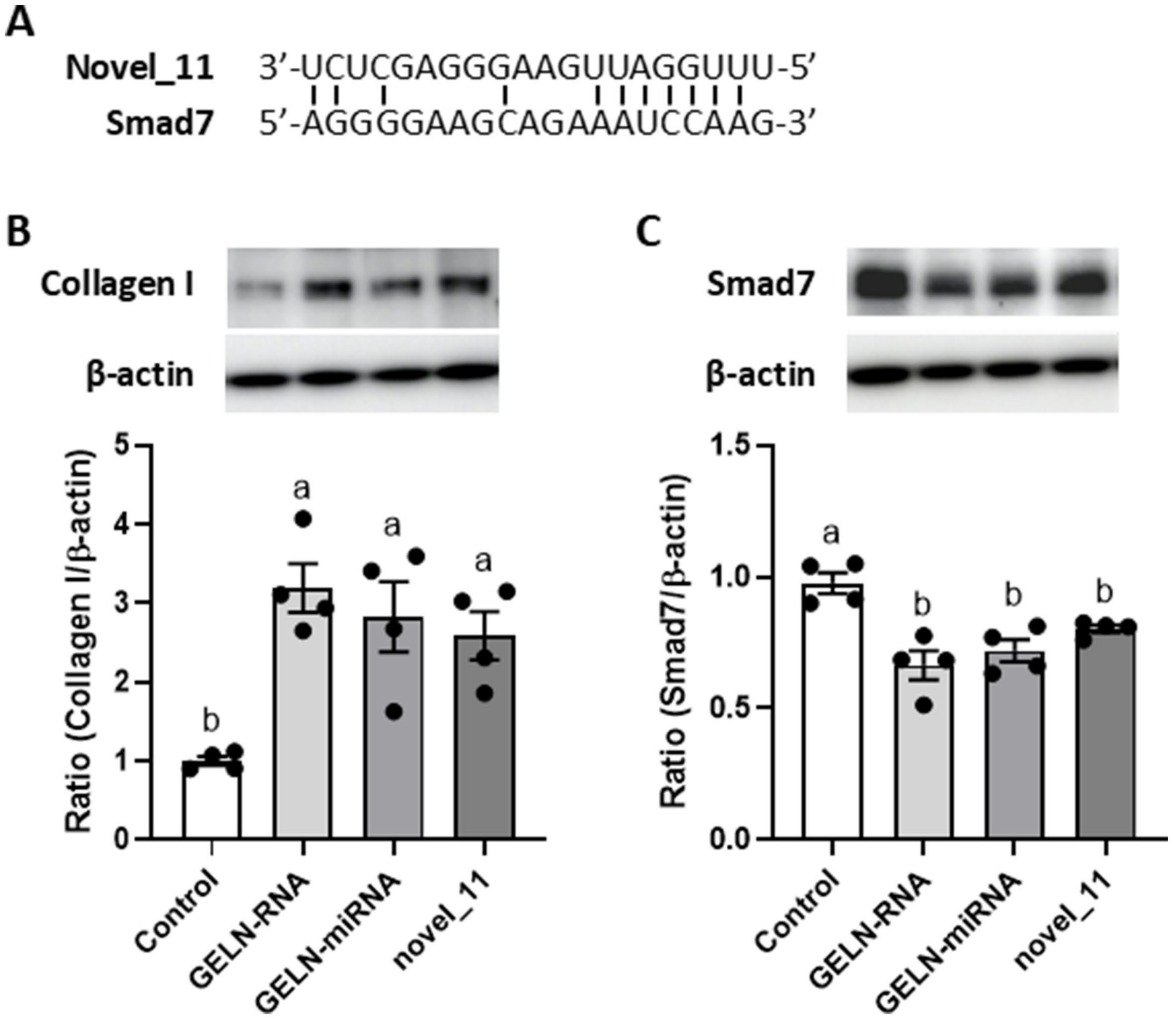
Effects of total RNA, miRNA-enriched fraction, and novel_11 miRNAs on collagen I and Smad7 expression in NB1RGB cells. **(A)** The sequence of putative novel_11 binding site in Smad7, **(B)** collagen I expression, **(C)** Smad7 expression. Values are presented as mean ± standard deviation (*n* = 4). Significant differences between groups were analyzed using one-way analysis of variance, followed by Tukey’s multiple comparison test. Different letters denote significant differences between the groups (*p* < 0.05).

We hypothesized that miRNA encapsulated within GELNs could enhance type I collagen expression by interacting with Smad7 mRNA. To test this hypothesis, we examined the effects of GELN-RNA and GELN-miRNA, on Smad7 expression. Additionally, next-generation sequencing was conducted to identify miRNA potentially binding to Smad7, followed by selecting a candidate for evaluation using a synthetic mimic RNA. Small RNA sequencing revealed that the most abundant small RNAs were approximately 20 nucleotides in length, identifying 12 novel miRNAs and one known miRNA within the GELNs small RNA libraries (Supplementary Figure 1 and Supplementary Tables 2, 3). Several of these miRNAs were complementary to Smad7, and among them, the novel miRNA with the highest transcript-per-million, designated as novel 11, was selected as a candidate. Figure 7A shows the sequence of novel_11 and a diagram of its putative binding site in Smad7. Type I collagen expression was significantly increased in cells transfected with GELN-RNA, GELN-miRNA, and the novel_11 mimic (Figure 7B). Furthermore, cells transfected with GELN-RNA, GELN-miRNA, and novel_11 exhibited a significant decrease in Smad7 expression (Figure 7C).

## 4 Discussion

Exosomes have important physiological functions, including the potential for intercellular communication. Similar to mammalian exosomes, plant-derived ELNs play functional roles, including cross-kingdom communication, and can target specific organs in animal models as delivery vehicles (18, 19). Plant-derived ELNs are extracted from various foods and herbs by juicing, ultracentrifugation, or ultrafiltration (18). We investigated the effects of GELNs isolated using ultracentrifugation on extracellular matrix component expression, particularly type I collagen, in NB1RGB cells. The TEM observations confirmed the presence of spherical GELNs, and the average particle size matched that of typical plant-derived ELNs, ranging from 30 to 400 nm (7). In NB1RGB cells, GELN treatment resulted in a dose-dependent increase in type I collagen expression, suggesting that GELNs effectively stimulate collagen production. Skin aging, the most visible sign of aging, is characterized by wrinkle formation, loss of elasticity, and uneven pigmentation (20). Maintaining skin texture relies on the integrity of the extracellular matrix, which involves the collagen framework. Wrinkle formation on the skin is associated with decreased collagen synthesis. Fibroblasts synthesize type I collagen as pre-procollagen, and cleaving the signal peptide leads to procollagen molecule formation (21). Procollagen is secreted by the cells and plays an important role in maintaining skin strength and elasticity. These results demonstrate that GELNs enhance collagen production, which may contribute to preventing skin aging.

To further investigate the bioactive compounds in GELNs, total RNA isolated from GELNs was transfected into NB1RGB cells, leading to increased mRNA expression of *COL1A1*. This suggests that the RNA within the GELNs may induce collagen synthesis. We performed subsequent microarray analysis to better understand the underlying mechanisms and potential impact of GELNs on cellular processes beyond collagen synthesis. GO microarray analysis collectively demonstrated that GELN-RNA-treated cells exhibit a robust activation of cell cycle processes and cell division, which are closely linked to increased collagen synthesis. This heightened cellular activity suggests active involvement in tissue growth and repair, where collagen production plays a fundamental role. These findings emphasize the importance of chromosomal dynamics, cytoskeletal organization, and regulatory mechanisms in supporting collagen synthesis. In addition, identifying energy-intensive pathways and protein–protein interactions underscores their essential role in the proper assembly and stabilization of collagen fibers within the extracellular matrix of treated cells. Microarray analysis revealed increased expression of genes associated with extracellular matrix formation. Based on these findings, we selected the relevant gene groups and validated their expression using qPCR. The results demonstrated that the expression of types IV and V collagen, elastin, hyaluronic acid synthases, hyaluronic acid-binding proteins, and TGF-*β*1 was also upregulated in addition to type I collagen. Types IV and V collagen contribute to stabilizing skin basement membrane structure, whereas elastin regulates skin flexibility and elasticity (21). Hyaluronic acid has a high water retention capacity, which maintains skin hydration and promotes elasticity. Furthermore, hyaluronic acid-binding proteins strengthen the extracellular matrix. TGF-β1 plays a crucial role in regulating collagen synthesis and extracellular matrix remodeling through the TGF-*β*/Smad signaling pathway (22). In this pathway, TGF-*β*1 activates Smad proteins, which translocate to the nucleus to regulate the expression of target genes involved in the production of collagen and other matrix components. This signaling cascade is essential for maintaining skin integrity, promoting wound healing, and controlling the balance between collagen synthesis and degradation. GELNs may enhance the activation of this pathway by upregulating TGF-*β*1, leading to increased production of types I, IV, and V collagen and other key extracellular matrix proteins, thereby contributing to improved skin structure and function.

The TGF-*β*/Smad signaling pathway plays a crucial role in collagen synthesis. TGF-β binds to its receptors to activate the TGF-β/Smad signaling pathway (23–25). In the cytoplasm, Smad2 and Smad3 typically exist as dimers, which become phosphorylated upon signaling. The phosphorylated Smad2 and Smad3 form a trimer with Smad4, which subsequently translocates to the nucleus to induce the expression of genes such as *COL1A1*. Smad7 acts as an inhibitory regulator by blocking the phosphorylation of Smad2 and Smad3, thereby negatively regulating the TGF-β/Smad signaling pathway (25). The most remarkable characteristic of plant-derived ELNs is their capacity to transport various nucleotides, including DNA, mRNA, miRNA, and non-coding RNA, between cells. In this study, we demonstrated that GELNs-miRNA enhances collagen production through the downregulation of Smad7. Among RNA molecules, miRNAs typically bind to specific mRNA to regulate their expression. This binding often occurs through imperfect base pairing rather than complete complementarity, allowing miRNAs to target multiple mRNAs, thus exhibiting relatively low substrate specificity. Given these characteristics, miRNA within GELN-RNA may play a key role in inhibiting Smad7 expression. Specifically, a mimic of novel_11, identified through small RNA sequencing and selected for its high abundance, promoted type I collagen expression by downregulating Smad7. Furthermore, among the other novel miRNAs identified, novel_2, novel_1, and novel_19 were also suggested to have potential complementary binding sites with Smad7, indicating their possible involvement in this regulatory process. These findings suggest that miRNA encapsulated in GELNs could effectively enhance type I collagen synthesis. While individual miRNAs in GELN-RNA are present at lower concentrations, it is likely that multiple miRNAs act synergistically to regulate Smad7 and promote collagen expression. Plant-derived ELNs generally contain many miRNAs; however, the types and numbers of miRNAs vary significantly between plant species (8). Therefore, characterizing the specific miRNA profiles within GELNs is crucial to understanding their role in promoting collagen synthesis. Differences across plant sources may impact their biological effects and specificity. Investigating the synergistic interactions among miRNAs within GELNs, as well as their dose-dependent effects, will provide deeper insights into the underlying mechanisms.

Many studies have demonstrated that plant-derived ELNs can withstand the harsh environment of the gastrointestinal tract, including exposure to stomach acid and digestive enzymes (7, 19). The lipid bilayer structure of ELNs may contribute to their stability, protecting their RNA and protein cargo during digestion. Additionally, previous research in mice has shown that orally administered acerola-derived ELNs were detected not only in the intestines but also in various tissues such as the brain, liver, bladder, and ovary (26). Tartary buckwheat-derived ELNs were detected in the liver and intestines following oral administration, suggesting their absorption and systemic circulation (27). To further enhance the applicability of GELNs, it will also be crucial to evaluate the effects of processing methods, such as spray-drying, on their integrity and functionality. Processing may influence the stability of the lipid bilayer and the cargo it protects, thereby affecting the bioavailability and physiological effects of GELNs. Additionally, ensuring their long-term stability under various storage conditions is essential for industrial applications, particularly in the fields of functional foods and pharmaceuticals. Further investigation concerning the stability of GELNs and the tissue distribution of orally administered GELNs will provide valuable insights into their bioavailability and potential physiological effects.

In conclusion, this study provides compelling evidence that ELNs derived from GEK juice and their RNA significantly influence collagen expression and extracellular matrix formation in human skin fibroblasts. The observed enhancement of type I collagen expression is likely mediated by the downregulation of Smad7 through miRNAs encapsulated within GELNs. These findings pave the way for further exploration of plant-derived ELNs for application in regenerative medicine and cosmetics. Given that there is no direct evidence regarding the transport of GELNs to skin tissues, further *in vivo* studies will be required to clarify the tissue distribution of GELNs. Additionally, understanding the influence of processing methods, such as spray-drying, on the stability and functionality of GELNs will be crucial for advancing their industrial applications.

## Data Availability

The raw data supporting the conclusions of this article will be made available by the authors, without undue reservation.
