# Supplementary material for: Exosome-like nanovesicles derived from kale juice enhance collagen production by downregulating Smad7 in human skin fibroblasts
Source: Front Nutr. 2025 Feb 10;12:1486572. doi: 10.3389/fnut.2025.1486572 (PMC11847687; doi:10.3389/fnut.2025.1486572)
Supplement: Supplementary file 1 [file Data_Sheet_1.docx]

Supplementary Material

Exosome-like nanovesicles derived from kale juice enhance collagen production by downregulating Smad7 in human skin fibroblasts

Peihan Hsu, Yuriko Kamijyo, Emiri Koike, Saki Ichikawa, Yifeng Zheng, Tomohiro Ohno, Shigeru Katayama*

*** Correspondence:** Shigeru Katayama (skata@shinshu-u.ac.jp)

**Supplementary Table 1.** Primers used in this study

| Gene | Primer sequence | | Product length (bp) |
| --- | --- | --- | --- |
| 18S rRNA | Forward | 5'-GTAACCCGTTGAACCCCATT-3' | 151 |
|  | Reverse | 5'-CCATCCAATCGGTAGTAGCG-3' |  |
| *COL1A1* | Forward | 5'-GAGGGCCAAGACGAAGACATC-3' | 140 |
|  | Reverse | 5'-CAGATCACGTCATCGCACAAC-3' |  |
| *COL4A1* | Forward | 5'-GGGATGCTGTTGAAAGGTGAA-3' | 113 |
|  | Reverse | 5'-GGTGGTCCGGTAAATCCTGG-3' |  |
| *COL4A2* | Forward | 5'-TTATGCACTGCCTAAAGAGGAGC-3' | 207 |
|  | Reverse | 5'-CCCTTAACTCCGTAGAAACCAAG-3' |  |
| *COL5A1* | Forward | 5'-GCCCGGATGTCGCTTACAG-3' | 80 |
|  | Reverse | 5'-AAATGCAGACGCAGGGTACAG-3' |  |
| *ELN* | Forward | 5'-GCAGGAGTTAAGCCCAAGG-3' | 148 |
|  | Reverse | 5'-TGTAGGGCAGTCCATAGCCA-3' |  |
| *TGFB1* | Forward | 5'-TACCTGAACCCGTGTTGCTCTC-3' | 122 |
|  | Reverse | 5'-GTTGCTGAGGTATCGCCAGGAA-3' |  |
| *HAS2* | Forward | 5'-CTCTTTTGGACTGTATGGTGCC-3' | 205 |
|  | Reverse | 5'-AGGGTAGGTTAGCCTTTTCACA-3' |  |
| *ITGA3* | Forward | 5'-TGTGGCTTGGAGTGACTGTG-3' | 139 |
|  | Reverse | 5'-TCATTGCCTCGCACGTAGC-3' |  |
| *VCAN* | Forward | 5'-GTAACCCATGCGCTACATAAAGT-3' | 110 |
|  | Reverse | 5'-GGCAAAGTAGGCATCGTTGAAA-3' |  |
| *HAPLN1* | Forward | 5'-TCTGGTGCTGATTTCAATCTGC-3' | 85 |
|  | Reverse | 5'-TGCTTGGATGTGAATAGCTCTG-3' |  |

**Small RNA sequencing and miRNA identification**

Small RNA sequencing and miRNA identification were conducted by Filgen (Nagoya, Japan) using the Illumina NovaSeq6000 platform. RNA samples (n=1) were isolated from exosome-like nanovesicles derived from glucoraphanin-enriched kale (GELNs). Raw reads in Fastq format were processed to remove low-quality reads and contaminants, resulting in clean reads. Small RNA tags were mapped to the reference sequence using Bowtie software for expression analysis. We identified known miRNAs by aligning the mapped tags with miRBase version 20.0, using miRDeep2 and srna-tools-cli for structure prediction and counting. Custom scripts were used to analyze base bias. We mapped sequences to the RepeatMasker and Rfam databases to exclude non-miRNA tags (e.g., protein-coding genes, rRNA, and tRNA). Novel miRNAs were predicted using miREvo and miRDeep2. Given secondary structure prediction of precursor sequences, novel miRNAs were identified, and all alignments were summarized, and unique small RNAs were assigned to a single category. miRNA editing and family classification were conducted using miFam data from miRBase or Rfam for novel miRNAs. miRNA expression was quantified as transcripts per million.

1. **Small RNA sequencing results**

GELN-RNA was subjected to small RNA sequencing. After removing impurities and low-quality reads, the GELN libraries yielded 20,223,473 clean reads (Supplementary Table 2). The most abundant small RNAs in both samples were approximately 20 nucleotides (Supplementary Figure 1A). The mapped small RNA reads from GELNs were 17,937,503. Small RNA annotation revealed several types of small RNAs in the samples, including miRNAs, rRNAs, tRNAs, and other small RNAs (Supplementary Figure 1B).

1. **Identification of known and novel miRNAs**

The reads mapped to the reference genome were compared with specific sequences using miRBase. We identified 12 novel miRNAs and only one known miRNA in the GELN small RNA libraries through small RNA sequencing (Supplementary Table 3).

**Supplementary Table 2.** Small RNA sequencing results from glucoraphanin-enriched kale-derived exosome-like nanovesicle libraries

| Sample | Total reads | Clean reads | Total sRNA | Mapped sRNA | “+” Mapped sRNA | “−” Mapped sRNA |
| --- | --- | --- | --- | --- | --- | --- |
| GELNs | 20,808,572 | 20,223,473 | 18,384,506 | 17,937,503 (97.57%) | 17,634,014 (95.92%) | 303,489 (1.65%) |

“+” Mapped sRNA: The number and percentage of mapped sRNA in the same direction as the genome.

“−” Mapped sRNA: The number and percentage of mapped sRNA in the opposite direction to the genome.

**Supplementary Table 3.** Small RNA Sequencing results for known and novel miRNAs in glucoraphanin-enriched kale-derived exosome-like nanovesicles.

| Name | Sequence (5'-3') | Count | TPM |
| --- | --- | --- | --- |
| Bol-miR172a | AGAAUCUUGAUGAUGCUGCAU | 2 | 8264.5 |
| novel_11 | UUUGGAUUGAAGGGAGCUCU | 117 | 483471.1 |
| novel_2 | UUUCCAAAUGUAGACAAAGC | 39 | 161157.0 |
| novel_1 | UUUUGGUGUGAGGAACUUAUCAUGA | 33 | 136363.6 |
| novel_17 | UUUGGAUUGAAGGGAGCU | 22 | 90909.1 |
| novel_5 | CUUCCGAUAAGAACUCCAUC | 8 | 33057.9 |
| novel_23 | UGAAUUAGACUGAGAAGA | 5 | 20661.2 |
| novel_21 | CUGUCUGUUAACUUUUAAC | 4 | 16528.9 |
| novel_22 | GUGUGUUGGGAAGAUCUCUAUG | 3 | 12396.7 |
| novel_16 | CUUCGGCUAGGAAAUCUAUCCU | 3 | 12396.7 |
| novel_4 | ACCCGUCUGUUAACUUUUAAC | 3 | 12396.7 |
| novel_19 | AUUUUUCCGUCGGAAUGUC | 2 | 8264.5 |
| novel_6 | CUUCCGAUAAGAACUCCACUCU | 1 | 4132.2 |

**Supplementary Figure 1.** Length distribution of the clean reads of glucoraphanin-enriched kale-derived exosome-like nanovesicles small RNA libraries (A) and distribution of RNA types. NAT, natural antisense transcript. The RNA categories displayed include known miRNA, novel miRNA, NAT (natural antisense transcripts), rRNA (ribosomal RNA), snoRNA (small nucleolar RNA), snRNA (small nuclear RNA), and tRNA (transfer RNA). Other RNA types, including exon-exon+, exon-intron+, intron-, intron+, and repeat sequences, are grouped under "other."
